# A multihost bacterial pathogen overcomes continuous population bottlenecks to adapt to new host species

**DOI:** 10.1126/sciadv.aax0063

**Published:** 2019-11-27

**Authors:** Rodrigo Bacigalupe, María Ángeles Tormo-Mas, José R. Penadés, J. Ross Fitzgerald

**Affiliations:** 1 The Roslin Institute, University of Edinburgh, Easter Bush Campus, Edinburgh EH25 9RG, UK.; 2 Centro de Investigación y Tecnología Animal, Instituto Valenciano de Investigaciones Agrarias, Segorbe 12400, Spain.; 3 Severe Infection Group of Instituto de Investigación Sanitaria La Fe, 106 Avenida Fernando Abril Martorell, Valencia 46026, Spain.; 4 Departamento de Ciencias Biomédicas, Universidad CEU Cardenal Herrera, Moncada 46113, Spain.; 5 Institute of Infection, Immunity and Inflammation, University of Glasgow, Sir Graeme Davies Building, 120 University Place, Glasgow G12 8TA, UK.

## Abstract

While many bacterial pathogens are restricted to single host species, some have the capacity to undergo host switches, leading to the emergence of new clones that are a threat to human and animal health. However, the bacterial traits that underpin a multihost ecology are not well understood. Following transmission to a new host, bacterial populations are influenced by powerful forces such as genetic drift that reduce the fixation rate of beneficial mutations, limiting the capacity for host adaptation. Here, we implement a novel experimental model of bacterial host switching to investigate the ability of the multihost pathogen *Staphylococcus aureus* to adapt to new species under continuous population bottlenecks. We demonstrate that beneficial mutations accumulated during infection can overcome genetic drift and sweep through the population, leading to host adaptation. Our findings highlight the remarkable capacity of some bacteria to adapt to distinct host niches in the face of powerful antagonistic population forces.

## Teaser

A model of bacterial host switching provides a window into the population dynamics of host adaptation.

## INTRODUCTION

Emerging infectious diseases often originate from pathogens that cross species boundaries to infect new host populations. For example, *Staphylococcus aureus* is an important human pathogen, but numerous host jump events have facilitated the emergence of endemic livestock strains (*1*, *2*) and subsequent host switches back into humans have led to the expansion of global epidemic clones (*3*–*5*). Accordingly, *S. aureus* represents a model pathogen to examine the bacterial traits associated with a multihost ecology. Understanding the genetic mechanisms of host adaptation is essential to identify novel molecular targets for tackling cross-species infections and preventing emerging clones. However, our knowledge of the population dynamics and host-adaptive genetic events that underpin these host-switching events is limited (*6*). To date, several studies have compared the genomes of contemporary clinical isolates from different host species and revealed genetic signatures associated with host adaptation (*2*, *5*, *7*). However, these traits reflect adaptation on a scale of decades to thousands of years, and the genetic events associated with adaptation in the days or weeks after a host-switch event remain poorly understood.

Following an interspecies host jump, bacterial populations occupying a new host may generate genetic diversity upon which selection can act to enhance fitness in the new host. However, the pathogen population can be affected by extreme population changes, such as those encountered in highly dynamic environments, which may result in bottlenecks (*8*, *9*). In addition, expansion into the new host species population requires transmission to other members of the new host species. During transmission between individuals, bacterial populations may undergo transmission bottlenecks that can cause drastic reductions in the population size (*10*). Events of this nature represent major evolutionary forces that can severely limit population diversity (*11*, *12*).

The impact of bottlenecks on adaptive evolution has been explored in theoretical frameworks (*12*–*15*), natural viral populations (*16*–*18*), and bacteria using in vitro experimental evolution (*11*, *19*–*22*). Populations influenced by periodic bottlenecks exhibit notably lower fixation rates of beneficial mutations due to genetic drift, thereby potentially limiting the capacity for host adaptation (*15*, *23*, *24*). However, the effect of within-host and transmission bottlenecks on the capacity of bacteria to adapt to a new animal host species has not been examined to date using an in vivo model.

Here, we develop a novel in vivo experimental model of a human to ovine host-switch event, involving the multihost pathogen *S. aureus*, providing the first opportunity to examine the adaptive evolution of a bacterial pathogen after a switch between natural host species. The infection model used is characterized by continuous within-host periodic and interhost transmission bottlenecks during experimental infections lasting up to 1 year. Both our integrated within-host population genomic approach and in silico evolutionary simulations indicated that the fitness gain of beneficial mutations is high enough to overcome the genetic drift imposed by continuous bottlenecks, allowing the adaptive mutations to sweep through the population. These data provide insights that help explain the capacity for *S. aureus* and other multihost pathogens to rapidly adapt to a new host species in the face of powerful antagonistic population forces.

## RESULTS

### Design of an experimental model of bacterial host switching

**Fig. 1:**
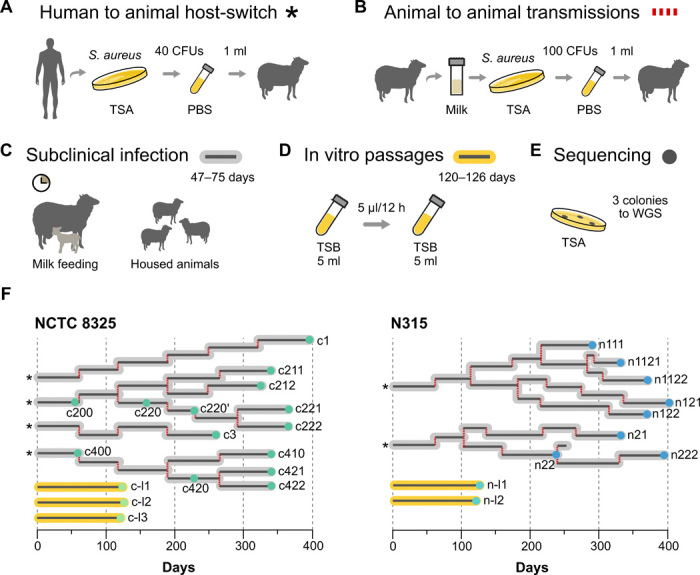
Experimental model of bacterial host switching and transmissions. (**A**) From the *S. aureus* human-associated parental strains, host switches were reconstructed by infecting ewes (represented with an asterisk). (**B**) Serial passages from sheep to sheep were performed every 3 to 5 weeks (red dashed lines). (**C**) Animals were housed with other infected animals and their lambs that frequently milk-fed from their mothers. (**D**) The parental human *S. aureus* strains were also passaged in vitro in nutrient-rich medium. (**E**) We selected three clones from some intermediate and the last isolation plates of every lineage for genomic DNA sequencing. (**F**) Representation of the entire transmission chains performed in the study for each strain.

To examine the adaptive evolution of *S. aureus* in the initial stages of a host switch, we developed a model of a human to sheep host-switch event (Fig. 1), as described in Materials and Methods. Briefly, we used two different human-associated *S. aureus* strains (NCTC8325 and N315) to establish subclinical infections of the mammary glands of ewes (Fig. 1A). The animals were housed in groups of infected ewes and their lambs, which carried out frequent milk feeding. The periodic reductions in intramammary milk volume due to feeding impose continuous bottlenecks on the intramammary bacterial population (Fig. 1C and Materials and Methods). Because successful host jumps (as distinct from spillover events) require the capacity to transmit to other individuals of the new host species (*25*), we also simulated subsequent sheep-to-sheep transmission events (Fig. 1B). For this purpose, milk samples obtained from infected sheep were grown on tryptic soy agar (TSA) plates, and multiple colonies of *S. aureus* isolated from the plates were used to infect additional animals. These passages were performed up to six or seven times in additional animals, leading to tree-form transmission chains with defined lineages and sublineages (Fig. 1F and table S1). In total, 156 sheep were infected, and the maximum infection duration time for a single lineage undergoing multiple passages was 400 days. Considering an approximate *S. aureus* replication time of 25 to 30 min (estimated from in vitro experiments), the infecting populations underwent the equivalent of approximately 18,000 to 24,000 generations (Fig. 1). In parallel, we carried out sheep intramammary infections with ovine-specialized clones of *S. aureus*, but the animals quickly developed severe infections with acute disease requiring antibiotic treatment, precluding their use to compare adaptive evolution of human and sheep strains in the current study. Therefore, as a control, we passaged human *S. aureus* strains in vitro in nutrient-rich medium (Fig. 1D).

### Genomic diversification of human *S. aureus* during infection passages in sheep

**Fig. 2:**
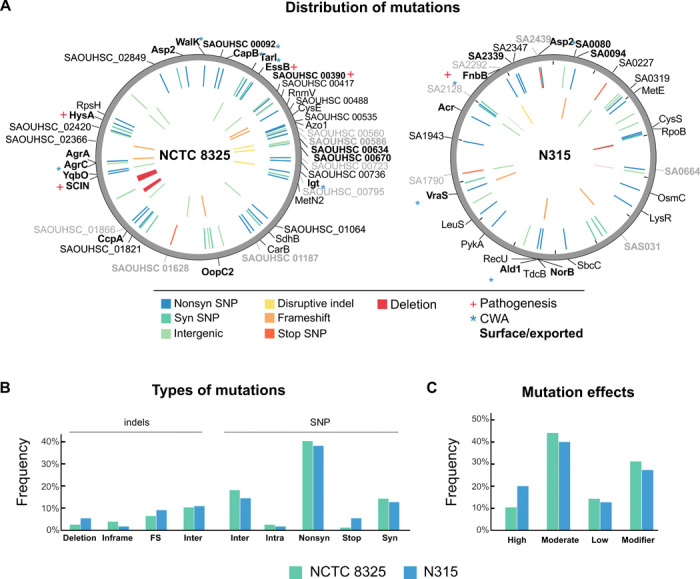
Mutations acquired during the infections and passages in sheep. (**A**) Distribution of different types of mutations across the genomes and genes affected, colored by type of mutation (bottom legend). SNPs are plotted in the outer circle, and indels are plotted in the inner circle. Protein products involved in pathogenesis and cell wall association (CWA) or that are exported/bacterial surface related are shown in bold. (**B**) Frequency of various types of mutations (FS: frameshift; Inter: intergenic; Intra: intragenic; Nonsys: nonsynonymous; Syn: synonymous). (**C**) Bar plots of frequency of mutation effects on the protein level (High: stop codon, frameshift; Moderate: nonsynonymous; Low: synonymous and intragenic SNPs) or in intergenic regions (modifier effect).

To examine the diversification of *S. aureus* during the course of infections after a host switch, we performed whole-genome sequencing (WGS) of three isolates from each of the primary isolation plates from the terminal-infected sheep (Fig. 1E). In addition, three colonies from isolation plates from five intermediate-infected sheep were sequenced (table S2). The lineages acquired single-nucleotide polymorphisms (SNPs), short indels, and large deletions with a random distribution throughout the genome (Fig. 2A). Of the 99 SNPs identified in total among the 51 isolates examined (27 isolates derived from NCTC8325 and 24 isolates derived from N315), 22 were in intergenic regions, 3 were intragenic, 52 were nonsynonymous, 18 were synonymous, and 4 led to disruption of genes by introducing premature stop codons. Of the indels, 10 resulted in frameshifts, 4 caused disruptive frame changes, and 14 were intergenic (Fig. 2B), with most mutations having moderate and modifier effects (Fig. 2C, Table 1, and table S3). Of the mutations that have impact on amino acid sequence, most were in genes encoding proteins involved in host-pathogen interactions, gene regulation, signal transduction, or transport and metabolism (Fig. 2A), including 47% affecting secreted proteins predicted to influence the cell wall envelope and/or pathogenesis (Table 1). For example, genes influencing capsule expression, the Esx secretion system, the staphylococcal complement inhibitor, the two-component regulators walK/R and vraS/R, accessory gene regulator (*agr*), and catabolite control protein A (*ccpA*) contained predicted amino acid replacements that could influence host-pathogen interactions. In addition to mutations, we detected large deletions in passaged isolates derived from both the NCTC8325 and N315 strains. For example, the prophage ϕ11 was excised during an early passage of NCTC8325 and was absent from all subsequent isolates, and a single isolate of NCTC8325 lacked a 28-kb region of the variable genome island νSaβ that contains an array of genes involved in host-pathogen interactions or bacterial competition.

**Table 1:** Mutations with moderate and high effect acquired during the infections and passages.

**Strain**	Mutation*	**bp**	**Type**	**Gene**	**Encoded product**	**Group**	**Lineage**
NCTC8325	SNP	27,460	Missense	*walK*	Sensor protein kinase WalK	GR | TM | ST	
NCTC8325	SNP	98,945	Missense	SAOUHSC_00092	Conserved hypothetical protein	HP | TM | SE	c222
NCTC8325	SNP	120,749	Missense	*cap5B*	Capsular polysaccharide synthesis enzyme Cap5B	SE | HP	
NCTC8325	SNP	24,5287	Missense	*tarI*	Conserved hypothetical protein	SE | HP	
NCTC8325	SNP	280,449	Missense	*essB*	ESAT-6 secretion machinery protein EssB	TM | SE | P	
NCTC8325	SNP	393,634	Missense	SAOUHSC_00390	Conserved hypothetical protein	HP | P	
NCTC8325	SNP	420,058	Missense	SAOUHSC_00417	Conserved hypothetical protein	TM	
NCTC8325	SNP	464,931	Missense	*rnmV*	Ribonuclease M5	TM	
NCTC8325	SNP	541,242	Missense	SAOUHSC_00535	Epimerase/dehydratase	TM	
NCTC8325	SNP	546,984	Missense	*azo1*	FMN-dependent NADPH-azoreductase	TM	
NCTC8325	SNP	568,990	Missense	SAOUHSC_00560	Conserved hypothetical protein		c222
NCTC8325	SNP	588,150	Missense	SAOUHSC_00586	Conserved hypothetical protein	SE	
NCTC8325	SNP	706,223	Missense	SAOUHSC_00723	Conserved hypothetical protein	TM	c222
NCTC8325	SNP	721,884	Missense	SAOUHSC_00736	Putative lipid kinase	TM	c222
NCTC8325	SNP	778,092	Missense	SAOUHSC_00795	Glyceraldehyde-3-phosphate dehydrogenase	TM | HP	c222
NCTC8325	SNP	813,228	Missense	*metN2*	Methionine import ATP-binding protein MetN2	TM	
NCTC8325	SNP	1,030,094	Missense	SAOUHSC_01064	Pyruvate carboxylase	TM	
NCTC8325	SNP	1,067,459	Missense	*sdhB*	Iron-sulfur subunit of succinate dehydrogenase%2C putative	TM	
NCTC8325	SNP	1,121,026	Missense	*carB*	Carbamoyl phosphate synthase large subunit	TM	
NCTC8325	SNP	1,138,999	Missense	SAOUHSC_01187	Conserved hypothetical protein	SE	
NCTC8325	SNP	1,550,169	Stop-gain	SAOUHSC_01628	Conserved hypothetical protein	SE	
NCTC8325	SNP	1,727,352	Missense	SAOUHSC_01821	Conserved hypothetical protein	TM	c222
NCTC8325	SNP	1,757,024	Missense	*ccpA*	Catabolite control protein A	GR | P	c222
NCTC8325	SNP	1,773,253	Missense	SAOUHSC_01866	Conserved hypothetical protein	TM	
NCTC8325	SNP	2,033,426	Missense	*scni*	Staphylococcal complement inhibitor	HP | P | SE	
NCTC8325	SNP	2,045,511	Missense	*yqbO*	Tail length tape measure protein		c222
NCTC8325	SNP	2,095,322	Missense	*agrC*	Accessory gene regulator protein C	GR | ST | P | SE	
NCTC8325	SNP	2,249,603	Missense	SAOUHSC_02420	Conserved hypothetical protein	SE	c222
NCTC8325	SNP	2,286,918	Missense	*hysA*	Hyaluronate lyase	TM | CWA | P	c222
NCTC8325	SNP	2,311,214	Missense	*rpsH*	Ribosomal protein S8	TM	c222
NCTC8325	SNP	2,624,646	Missense	SAOUHSC_02849	Putative pyruvate oxidase	TM	
NCTC8325	SNP	2,756,289	Missense	*asp2*	Accessory Sec system protein Asp2	TM | SE	
NCTC8325	Ins	487,126	Frameshift	SAOUHSC_00488	Cysteine synthase	TM	
NCTC8325	Del	513,207	Frameshift	*cysE*	Serine acetyltransferase	TM	
NCTC8325	Del	624,930	Disruptive	SAOUHSC_00634	ABC transporter	TM | SE	
NCTC8325	Ins	658,822	Disruptive	SAOUHSC_00670	Conserved hypothetical protein	TM | SE	c222
NCTC8325	Del	764,412	Disruptive	*lgt*	Prolipoprotein diacylglyceryl transferase	TM | SE	
NCTC8325	Del	1,323,611	Frameshift	*oppC2*	Oligopeptide transporter putative membrane permease domain	TM | SE	
NCTC8325	Del	2,096,058	Frameshift	*agrA*	Accessory gene regulator protein A	GR | ST | P	
NCTC8325	Ins	2,188,845	Frameshift	SAOUHSC_02366	Conserved hypothetical protein	TM	
N315	SNP	105,983	Missense	SA_RS00630	Lipoprotein	SE	
N315	SNP	274,258	Stop-gain	SA_RS01330	Acetyl-CoA/acetoacetyl-CoA transferase	TM	n222
N315	SNP	377,083	Missense	SA_RS01835	PTS lactose transporter subunit IIB	TM	
N315	SNP	403,331	Missense	*metE*	5-Methyltetrahydropteroy-ltriglutamate— homocysteine methyltransferase	TM	
N315	SNP	572,540	Missense	*cysS*	Cysteine—tRNA ligase	TM	
N315	SNP	580,166	Missense	*rpoB*	DNA-directed RNA polymerase subunit beta	TM	
N315	SNP	758,669	Missense	SA_RS03785	Hypothetical protein	SE	
N315	SNP	863,049	Missense	*osmC*	Organic hydroperoxide resistance protein	HP	
N315	SNP	949,546	Missense	*LysR TR*	LysR family transcriptional regulator	GR	
N315	SNP	1,343,567	Missense	*sbcC*	Nuclease SbcCD subunit C	TM	
N315	SNP	1,471,383	Missense	*norB*	Quinolone resistance protein NorB	TM | P | SE	
N315	SNP	1,486,091	Missense	*recU*	Holliday junction resolvase RecU	TM	
N315	SNP	1,735,479	Missense	*pykA*	Pyruvate kinase	TM	n222
N315	SNP	1,817,943	Missense	*leuS*	Leucine—tRNA ligase	TM	
N315	SNP	1,948,268	Missense	*vraS*	Two-component sensor histidine kinase	GR | ST | SE | P	
N315	SNP	2,035,898	Stop-gain	SA_RS10285	Hypothetical protein		n222
N315	SNP	2,193,397	Missense	SA_RS11160	EVE domain–containing protein	TM	
N315	SNP	2,320,771	Missense	*acr*	AcrB/AcrD/AcrF family protein	TM | SE	
N315	SNP	2,393,851	Missense	SA_RS12225	MOSC domain–containing protein	TM	
N315	SNP	2,575,229	Missense	SA_RS13145	Hypothetical protein	CWA | HP	
N315	SNP	2,626,125	Missense	SA_RS13405	Membrane protein	SE	
N315	SNP	2,637,600	Missense	SA_RS13450	*N*-succinyldiaminopimelate aminotransferase	TM	n222
N315	SNP	2,743,605	Stop-gain	SA_RS13980	Hypothetical protein	CWA | SE	
N315	SNP	2,751,067	Missense	*asp2*	Accessory Sec system protein Asp2	TM | SE	n222
N315	Del	90,901	Frameshift	SA_RS00555	Anion membrane transporter	TM | SE	
N315	Del	1,085,977	Frameshift	SA_RS05425	Hypothetical protein	SE	
N315	Del	1,473,471	Frameshift	*tdcB*	l-Threonine dehydratase catabolic TdcB	TM	
N315	Ins	1,475,240	Frameshift	*ald1*	Alanine dehydrogenase	TM | CWA | SE	
N315	Del	1,947,711	Frameshift	*vraS*	Two-component sensor histidine kinase	GR | ST | P | SE	
N315	Del	2,568,566	Disruptive	*fnbA*	Fibronectin-binding protein A	CWA | HP | SE | P	

*Only mutations with moderate effect (missense SNPs and disruptive indels) and high effect (stop-gained and frameshifts) are listed because they are more likely to play a role in host adaptation. Mutations include SNPs, deletions (Del), and insertions (Ins). Group: HP (host-pathogen interaction), GR (gene regulation), ST (signal transduction), TM (transport and metabolism), P (pathogenesis), CWA (cell wall associated), and SE (surface associated/exported). Lineage indicates the fittest clone in which mutations were identified.

In addition, lineage N2 lost the *sdr* gene cluster, encoding serine-aspartate repeat-containing proteins that mediate interactions with human epithelial or innate immune cells (*26*, *27*). Of note, although the experiment was designed to allow the potential acquisition of mobile genetic elements (MGE) from resident sheep bacteria, possibly mediated by lambs feeding on multiple ewes, none was identified, suggesting robust barriers to genetic acquisition or isolation from potential reservoirs of MGE.

For in vitro passaged lineages, isolates accumulated more mutations, but there was greater variation between lineages (6 to 23 mutations). SNPs were primarily nonsynonymous affecting genes encoding enzymes involved in metabolism, including nonsense mutations associated with loss of gene function (Table 1). In addition, several phages and a 17-kb cluster of genes involved in the biosynthesis of staphyloxanthin were deleted. These data indicate several metabolic pathways that are dispensable for survival under nutrient-rich conditions in vitro (*28*).

### Genomic signatures of population bottlenecks linked to loss of diversity

**Fig. 3:**
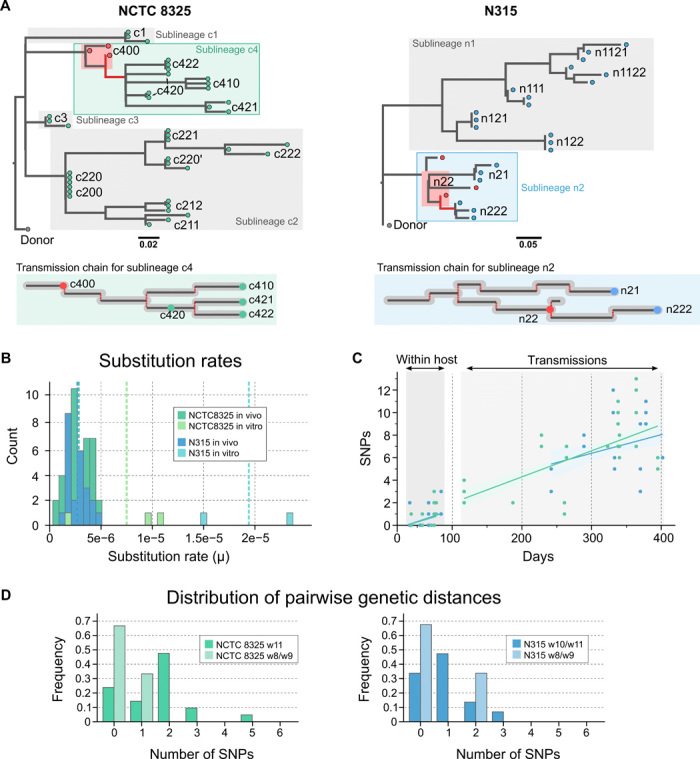
Evolutionary dynamics of infection passages. (**A**) Minimum evolution trees of the passages constructed using SNP data only are consistent with the topologies and lineages of the transmission chains. Differences in branch lengths for isolates from specific sheep indicate the coexistence of different alleles within the animals, supporting genetic variability arisen from single clones after a transmission. Clones c422 and c221 also presented some indels not represented in these trees. (**B**) Lineages from which intermediate isolates were sequenced are marked in red. Substitution rates for in vivo and in vitro passages of the strains N315 and NCTC8325. (**C**) SNP accumulation over time during transmissions and/or within host only. (**D**) Distribution of pairwise genetic distance between weeks 8 and 9 (early) and weeks 10 and 11 (late) indicates late accumulation of diversity within hosts.

We constructed minimum evolution phylogenetic trees for all the isolates derived from each sheep passage experiment based on the core SNPs (Fig. 3). Tree topologies were congruent with the transmission chains generated during the infection experiments (Fig. 1). Depending on the rate at which new mutations arise and the fitness benefit effect of those mutations, within-host evolution may reflect dominance of a single strain at any time point due to periodic selection or coexistence of multiple genotypes within the host due to clonal interference (*29*). Considering the effects of transmission between individuals, we postulate three possible scenarios: (i) dominance of a single strain before and after transmission; (ii) diverse genotypes coexisting within individuals, but only a single clone dominating after transmission; and (iii) diverse genotypes coexisting within a host and after transmission. Examination of the topology of the phylogenetic trees (Fig. 3A) supports the proposed scenario (ii), where different branches representing multiple isolates from individual sheep indicate the accumulation of genetic diversity within those hosts, followed by dominance of a single lineage after a passage to a new host (Fig. 3A). This is further supported by sequencing of isolates from intermediate time points of the passaging process, which represent distinct subbranches of the tree (Fig. 3A, red nodes). Nevertheless, in three cases, the isolates from the sheep were indistinguishable, which indicates that although the clone dominating after the transmission usually diversifies, it may also result in a clonal population (c220, n121, and n122). Differences in the branch lengths of minimum evolution trees indicate that distinct genotypes accumulate mutations at different rates.

### Population diversity is reduced during experimental host switches

During the sheep passages, bacteria exhibited an average substitution rate of 2.78 mutations per million base pairs (Mbp) per year (Fig. 3A), analogous to previous estimates for *S. aureus* in humans (*30*). The estimated rates for independent lineages were normally distributed, and there was no evidence for hypermutators, which have been previously linked to host adaptation (*31*). *S. aureus* cultured under nutrient-rich conditions in vitro had an average substitution rate of 12.3 SNPs/Mbp per year, a rate 4.5 times higher than the in vivo rate (Fig. 3B). We can attribute this difference to the higher replication rate of *S. aureus* in nutrient broth compared to intramammary infections (see Materials and Methods), where suboptimal environmental conditions and the host immune response slow bacterial growth.

To further explore the within-host dynamics and the impact of transmissions on genetic diversity, we plotted the number of SNPs accumulated in all sequenced isolates versus the total number of infection days and fitted linear models (Fig. 3C). Although the graph shows a high dispersal of SNPs due to variation of mutations in independent lineages, it indicates a strong temporal correlation and the molecular clock line crosses the *x* axis around 0 for the transmitted isolates (*a* = 0.021), consistent with the use of an initial inoculum represented by a single genotype. In contrast, when we only plotted SNPs identified among isolates from a single animal, trend lines crossed the time axis at over day 33 (*a* = 0.019, *b* = 0.636), consistent with a delay in the appearance of genetic diversity (Fig. 3C). Because the rate at which new mutations arise in the populations is constant (Fig. 3), the absence of diversity in the time after the initial inoculation/transmission simulation can be explained by recent fixation of genotypes due to genetic drift or selection of beneficial mutations. This observation is further supported by the distributions of pairwise genetic distances between isolates sampled from within individual hosts at different time points (8 to 9 weeks or 10 to 11 weeks) (Fig. 3D). At earlier time points, infections from clonal populations follow a geometric distribution, indicating no or little genetic variation between pairs of isolates, which turns into a geometric Poisson approximation as time proceeds and mutations are accumulated (*32*). The low genetic diversity observed at week 8 after inoculation is explained by either the continuous bottlenecks purging the accumulated population diversity, in part due to frequent reductions in milk volume caused by feeding lambs, or a recent sweep through the population of a beneficial mutation. The data also indicate population bottlenecks associated with transmission to a new host and subsequent stochastic expansion of a subpopulation of the infecting inoculum.

### Experimental infection passages lead to enhanced fitness in the new host species

**Fig. 4:**
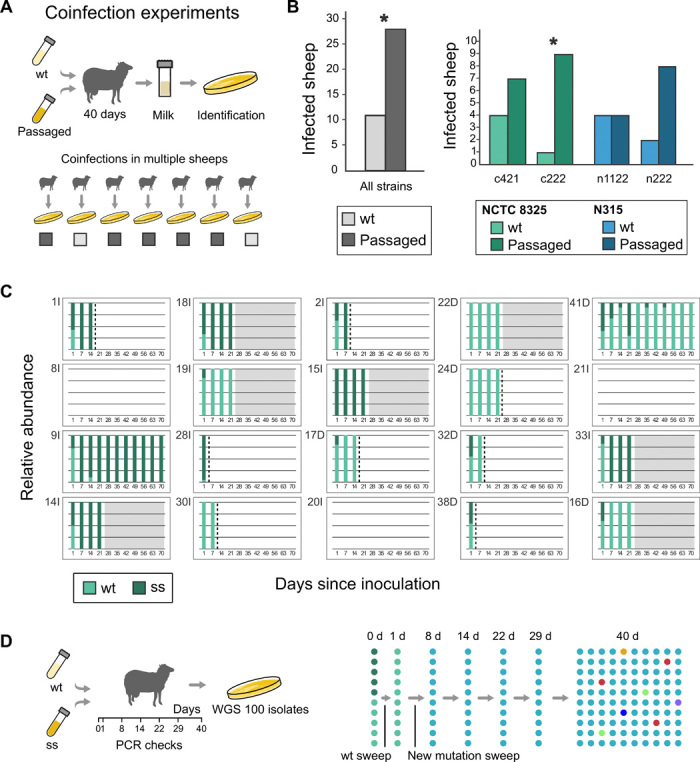
*S. aureus* has undergone adaptive evolution during passage in a new host species. (**A**) Competition experiments were performed by coinfecting sheep with equal number of progenitor and passaged bacteria derived from wild-type (wt) and passaged strain, and 40 days later, infected sheep were assessed for strain carriage. (**B**) Significant differences were obtained when compared to the 50-50% outcome expected for a null hypothesis of no adaptation (*P* = 0.039, one-tailed Fisher’s exact test; *P* = 0.027, Barnard’s test). (**C**) Proportions of the wt strain (green) and the strain with a synonymous SNP (ss) for coinfection experiments of 20 sheep. Dashed lines indicate the last time bacteria were isolated from milk. (**D**) WGS of 100 isolates after coinfection revealed the within-host genetic variation.

We next examined the impact of the bottlenecks on the passaged populations and investigated whether the apparent genetic loss observed in our model system was due to genetic drift or natural selection. We performed competition experiments (*33*) by coinfecting sheep with an inoculum composed of an equivalent number of progenitor and passaged bacteria derived from either strain N315 or NCTC8325 (Fig. 4A). After 40 days, bacteria were recovered from infected sheep, and in all cases, only one of either the progenitor or passaged genotypes was isolated, consistent with narrow bottlenecks in the early stages of infection. Notably, for 28 of the 39 sheep coinfected (72% of cases), the passaged strain was recovered more frequently than the human progenitor strain (Fig. 4B), indicating that *S. aureus* evolved enhanced fitness during the experimental infection passages (*P* = 0.0394, one-tailed Fisher’s exact test; *P* = 0.027, Barnard’s test). For only a single progenitor-passaged strain pair, there was no difference in the number of times the passaged strain was recovered in comparison to the progenitor. To examine whether the model selected for increased adaptation to milk or infectivity, we compared the growth of the two most successful strains (n222 and c222) in sheep milk to their respective progenitors. Although the passaged strains grew slower than the original, the differences were not statistically significant (fig. S2). Of note, in control experiments where competition infections were carried out between *S. aureus* strains of human and ovine clonal origin, only the ovine strains were recovered, highlighting the fact that ovine strains are highly adapted to the sheep mammary gland and can readily outcompete unadapted strains (table S4). Overall, these data demonstrate that, despite severe stochastic bottlenecks, *S. aureus* can rapidly evolve enhanced fitness in the new host species.

Competition experiments with isogenic strains of equivalent fitness (differing only by a single synonymous mutation in a pseudogene; NCTC8325 and NCTC8325s) demonstrated that the bottlenecks were a stochastic process, and in the absence of a fitness advantage, there is an equivalent likelihood of either strain outcompeting the other (Fig. 4C). From each of the 20 colonized animals, NCTC8325 and NCTC8325s genotypes were recovered at a frequency of 8:8 before establishment (grayed out) or clearance (dashed line). Examination of relative abundance of the coinfecting genotypes revealed the coexistence at day 1 in almost all cases, but only a single genotype was recoverable by day 5 (Fig. 4B and table S5). In addition, we used this experimental setup to provide a high-resolution picture of the within-host population dynamics. For this purpose, we sequenced 100 *S. aureus* isolates representing colonies isolated from a sheep 40 days after coinfection with NCTC8325 and NCTC8325s (Fig. 4D). Among the 100 isolates examined, 6 SNPs were identified, of which only 1 nonsynonymous SNP was fixed in the population [in the locus *aacA*, encoding the acetyl–coenzyme A (CoA) carboxylase α subunit]. Additional screening of 10 colonies isolated every week after the initial inoculation revealed that the mutation achieved fixation during the first week after inoculation (table S7). The other five SNPs were present in very low frequency, representing 1 to 3% of the population. Screening for the SNP found in 3% of the population (missense variant in hypothetical protein) did not identify the mutation among 400 colonies isolated at 14, 22, and 29 days, indicating that it occurred during the last 12 days of the infection (table S7). Together, these data are consistent with our previous analysis, indicating that bottlenecks limit the fixation of mutations by purging of the accumulated variation due to genetic drift.

### Identification of molecular correlates of host adaptation

To investigate the genetic basis for the observed enhanced fitness, we searched for host-adaptive genetic signatures or genes that acquired similar mutations in independent lineages, indicating convergent or parallel evolution. The *asp2* gene encoding a component of the secretory system responsible for the export of serine-rich glycoproteins to the bacterial surface (*34*) contains nonsynonymous mutations in two lineages derived from the N315 and NCTC8325 strains, respectively. Of note, the passaged strain n222 containing the *asp2* gene mutation presented one of the highest levels of fitness in comparison to its progenitor strain in the coinfection experiments (recovered in 80% of cases). In addition, n222 and the most fit passaged strain (c222; Fig. 4B) presented the largest proportions of nonsynonymous to synonymous SNPs (9/1 and 4/0, respectively), including several associated with genes encoding proteins that affect host-pathogen interactions (Table 1).

### Beneficial mutations emerge in the face of regular bottlenecks

**Fig. 5:**
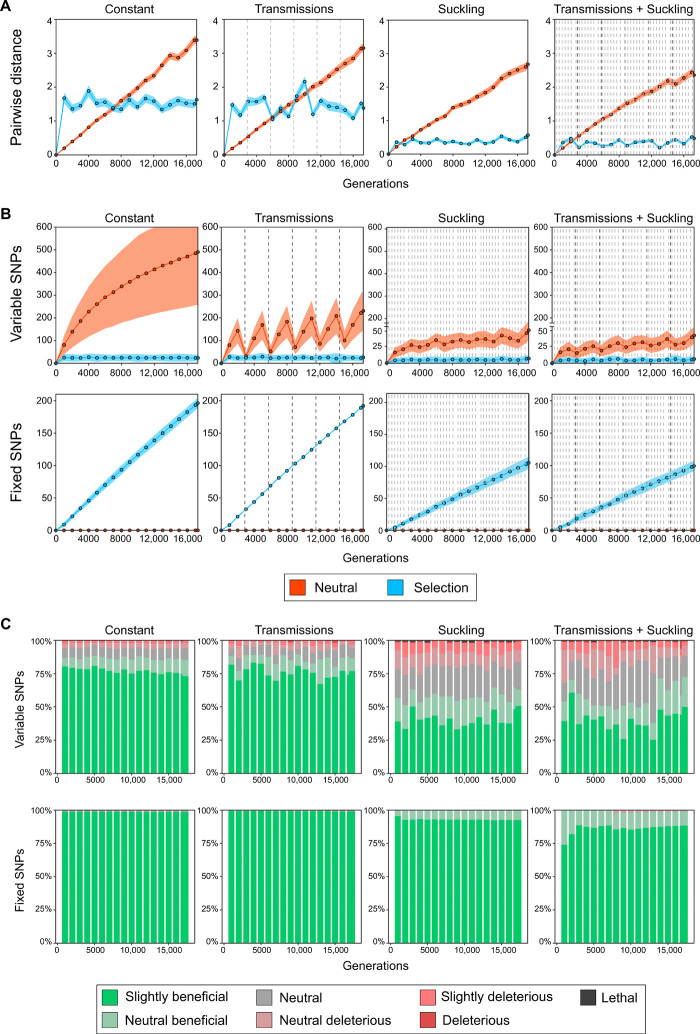
Simulations of genomic populations under transmission and feeding bottlenecks. (**A**) Average pairwise genetic distances between randomly selected isolates from the populations simulated. (**B**) Accumulation of fixed and variable SNPs over time. (**C**) Types of variable and fixed SNPs determined from the selection coefficients associated with every nucleotide.

To quantify the impact of transmission and within-host bottlenecks on the genomic diversity and the nature of mutations selected, we carried out simulations of models of the evolution of bacterial genomes using a forward time simulation model (*35*). Contrary to other in silico evolution experiments, the parameters used in our model replicated the initial natural conditions of our experimental design, and we let the populations evolve, allowing us to compare computational simulations with experimental observations. To account for the differences between the different bottlenecks, we reproduced four scenarios: (i) a constant population size, (ii) transmissions between individuals, (iii) feeding bottlenecks, and (iv) a combination of both. Using this software, we specified the selection coefficients for every mutation in the genome and simulated two models, one with all neutral mutations and another with selection coefficients following a hypothetical previously determined distribution (Fig. 5 and Materials and Methods) (*29*, *36*). Simulations were run for more than 17,000 generations, similar to the estimated replications for the in vivo experiments, with sampling of 100 isolates every 1000 generations. Pairwise genetic distances increased over time for the neutral model and remained constant when selection occurred, indicating that in the absence of selection, mutations accumulate steadily in the population (Fig. 5A). In both scenarios, bottlenecks resulted in a reduction of the population diversity. Next, we looked at the accumulation of variable and fixed SNPs over time (Fig. 5B). In the absence of selection, variable SNPs increase logarithmically toward an equilibrium, consistent with the pairwise genetic distances observed. As expected, bottlenecks considerably reduced the number of variable SNPs but still allowed some genetic variation to remain in the population without ever reaching fixation. However, once we introduced selection (as expected during our model of host switching), some mutations swept through the population and became fixed, causing a drastic reduction in the number of variable SNPs. The number of SNPs that became fixed in the simulations was higher than in our in vivo experiments, possibly because of differences between the model and the experimental infections in relation to the size of the bottlenecks, mutation rates, the generation time within sheep, or the selection coefficient distribution.

Last, we determined the types of coefficients associated with the variable and fixed SNPs in the four scenarios (Fig. 5C). Within a host, variable SNPs represent the diversity on which selection acts to fix them into the population. As expected, in the absence of bottlenecks, beneficial mutations outcompete neutral and deleterious SNPs and tend to accumulate over time. Although deleterious and lethal mutants are rapidly purged, transmission and feeding bottlenecks increase the power of genetic drift, leading to an accumulation of neutral and deleterious SNPs, which, after natural selection, leave some neutral mutations remaining. Of note, during the competition coinfection experiments, one of the four lineages had an indistinguishable level of fitness compared to the ancestral strain, suggesting that the accumulated mutations were neutral. Together, our experimental observations and the simulations support the capacity for beneficial mutations to rapidly spread through the population and become fixed, even in the presence of highly antagonistic population forces.

## DISCUSSION

Transmission of bacterial pathogens to a new host species may represent transient spillover events that result in dead-end infections (zoonoses) (*37*) or may lead to onward transmission to other individuals of the new host species (host jumps) (*25*). In the current study, we designed a novel infection model to study bacterial population dynamics during a host-jump event. Our model represents the first to allow experimental evolutionary analysis of a host switch between natural host species. Using this approach, we found that each transmission to a new host resulted in a bottleneck followed by an infection founded by a tiny fraction of the transmitted population that may even be represented by a single bacterial cell. Other models have reported similar phenomena for murine intravenous infections by *Streptococcus pneumoniae* (*38*) and murine or zebrafish infections experimentally infected with *S. aureus* (*39*). During these scenarios, the power of genetic drift is so overwhelming that beneficial mutations can be easily purged (*40*), and consequently, deleterious and neutral mutations may reach fixation in higher proportions due to the process known as the Muller’s ratchet effect (*41*). However, in our model system designed to examine the capacity for host-adaptive evolution, we found that most passaged strains acquired enhanced fitness during the infection experiments. In support of these findings, in silico evolution analysis predicted that beneficial mutations increase over time even when bottlenecks are frequent, suggesting that the fitness gain of beneficial SNPs is high enough to overcome genetic drift and sweep through the population. When the population size is constant, multiple beneficial mutations are expected to arise, and competition may slow down their progression toward fixation. However, although continuous bottlenecks produce random genetic drift and beneficial mutation loss, as a new population emerges from very few individuals, beneficial mutations can rapidly achieve fixation. The power of natural selection to overcome genetic drift and preserve genetic variation has been observed in natural populations of commensal gut bacteria (*42*).

An array of mutations that may affect host-pathogen interactions was identified including some in genes encoding proteins involved in pathogenesis. Of note, the polymorphisms in gene regulators may lead to remodeling of global regulatory networks, which influence the metabolic and pathogenic fitness of bacteria (*43*, *44*). Although gene acquisitions were not observed to occur during infection, the large deletions we detected for some lineages are consistent with gene loss as a mechanism for host adaptation (*4*, *7*, *45*, *46*). Other studies of bacterial within-host evolution have identified large-scale deletions more frequently than gene acquisition (*47*). We originally infected several sheep with ovine-adapted strains carrying MGE that we hypothesized could be transmitted to the human-associated strains and facilitate host adaptation (*7*). However, these animals quickly developed severe mastitis and were discarded from the study following treatment with systemic antibiotics. We did not determine the effect of individual mutations in the host-adaptive process, and (with the exception of *asp2*) the lack of observed convergent evolution in different lineages suggests that multiple pathways of host adaptation are likely possible.

In conclusion, the experimental infection model developed here represents an excellent system to understand host-adaptive evolution, affording a high-resolution insight into the population dynamics during the critical stages after a host-jump event. The model is characterized by frequent within-host and transmission-associated bottlenecks that cause severe genetic drift. Although we aimed to replicate the natural routes for transmission, their frequency and the amount of bacteria transferred between hosts may differ from those occurring during natural infections. In addition, we consider our model of a host switch to be an infection model as it requires introduction of bacteria to the generally germ-free environment of the mammary gland, leading to the establishment of a subclinical infection. It is important to note that a different type of model of host switching involving colonization only (for example, of the skin) would likely result in very different selective pressures affecting host adaptation and the evolutionary trajectory of bacterial host adaptation may be quite different.

Despite the existence of powerful antagonistic evolutionary forces that purge the population of diversity, our findings demonstrate that bacteria can undergo host-adaptive evolution leading to enhanced fitness in the new host species. The observed gain of beneficial mutations in the face of repeated bottlenecks and genetic drift highlights the remarkable capacity for some bacteria to adapt to new host species.

## MATERIALS AND METHODS

### Experimental design, sheep infections, and in vitro passages

We simulated *S. aureus* host jumps by inoculating the mammary gland of sheep with two human-associated strains. For these experiments, we used two very well characterized strains, NCTC8325 and N315, with high-quality genomes with well-curated genes and representing different successful clonal complexes of *S. aureus* diversity. In addition, these strains have undergone very limited passaging. N315 was obtained from the original stocks (K. Hiramatsu), while the sigma restored version of strain NCTC8325 was derived from the original stocks of R. Novick. For the initial inoculations, 40 colony-forming units (CFUs) from overnight cultures on TSA plates were suspended in 1 ml of phosphate-buffered saline and injected into the teat ducts. Sheep were kept isolated from their lambs for 4 hours after inoculation to prevent loss of bacteria due to suckling but then were released into groups with other infected ewes and their lambs. A few days after the inoculation, some ewes developed subclinical intramammary infections, while others cleared the bacteria. We milked infected sheep every day, and after 47 to 75 days, we simulated transmission by passing bacteria to new sheep. For these passages, 1 ml of the milk sampled was grown on TSA plates, and 50 to 100 colonies were picked from these primary isolation plates and used to infect new animals as described before, infecting with 100 CFUs. This process was repeated for 165 to 400 days, depending on the strain and success of the infections, representing four to seven passages for each lineage (table S1 and Fig. 1). The within-host bottlenecks imposed were assumed, considering the continuous feeding by lambs such that ewes had to be separated from the lambs for 2 to 3 hours before sampling so that enough milk could be collected. For this reason, and for the highly variable bacterial counts (50 to 5 × 10^3^ CFU/ml) obtained from sequential milk samples, the bottlenecks imposed by feeding were not quantifiable. From the last isolation plates obtained from the milk samples, each of the three colonies was picked for WGS (table S2). In vitro passages of strains NCTC8325 and N315 were performed by preparing cultures in tryptic soy broth (TSB) and transferring 5 μl of each culture into 5 ml of fresh medium every 12 hours. Passages were performed for 120 to 126 days, bacterial dilutions were plated onto TSA medium, and single colonies were picked for WGS (table S2).

### Competition experiments of parental and passaged strains

Additional sheep were coinfected with equal numbers of progenitor and passaged strains (1000 CFUs in total) following the procedure described above (table S4). We introduced a single restriction site synonymous mutation in the noncoding region of a pseudogene in the progenitor strains: the *vWbp* pseudogene in NCTC8325 and the *arlR* pseudogene in N315. These mutations were used to track the abundance of progenitor and passaged strains in the milk sampled from the coinfected ewes. From the isolation plates, at least 10 colonies were sampled for polymerase chain reaction (PCR) amplification and restriction enzyme digestion of the relevant genomic region. In addition, two isogenic strains, NCTC8325 and NCTC8325s (synonymous SNP in the *vWbp* pseudogene), were used to coinfect 20 additional sheep by inoculating 1000 CFUs of each of their mammary glands. For every week during 10 weeks, PCR amplification and restriction digestion with Eco RI were performed on 10 colonies isolated from the milk samples.

### Genomic sequencing, assembly, and annotation of genomes

Genomic DNA from overnight cultures of *S. aureus* isolates was extracted using the PurElute Bacterial Genomic Kit (Edge Biosystems, MD) with modification as previously described (*4*). Illumina libraries were prepared with the Nextera XT Kit for both MiSeq and HiSeq sequencing at Edinburgh Genomics. Two paired-end sequencing runs were produced for every isolate, obtaining reads of 100 nucleotides (nt) (HiSeq) or 200 nt (MiSeq). Quality control was performed using FastQC, adapters in reads were counted using the count_barcodes_by_kmer.pl scripts, and top adapters and low-quality reads were trimmed using Trimmomatic (*48*). De novo genomic assemblies were performed using SPAdes 3.8 (*k* values of 21, 33, 55, 77, 99, and 127) (*49*), resulting in a median of 67 contigs per genome (32 to 237 contigs) with an average *N*_50_ value of 199 kb (28 to 817 kb). Genomes were annotated using Prokka1.11 (*50*) with the -usegenus *Staphylococcus* option.

### Identification of genomic variants: SNPs, deletions, and insertions

Sequencing reads of the isolates were mapped to their respective reference genomes [NCTC8325, NC_007795 (*51*) and N315, NC_002745 (*52*)] using BWA with default parameters (*53*). SNPs and small indels were identified using the Genome Analysis Toolkit (*54*) and Picard Tools (https://broadinstitute.github.io/picard/). In GATK, we used the indel realignment and base recalibration options, and variants were recalibrated before filtering, discarding those with QualByDepth below 2.0, RMSMappingQuality below 40, and PHRED quality below 30. The genetic variants with a depth below 5 or not supported by 80% of the reads were filtered out, and the identified variants were manually checked in the alignment files to ensure that only true positives were included. The annotation of genetic variants and the prediction of their effects were performed with SnpEff (*55*). Medium-sized indels (few tens or hundreds base pairs) were identified using Pindel (*56*) and large deletions (>1 kb) with the coverageBed utility of BEDtools (*57*) by splitting the reference genome into windows of 1 kb that were then scanned in search of those presenting at least 500 bp with zero coverage. We searched for potential acquisition of MGEs and long insertions by assembling unmapped reads and running BLAST searches of contigs longer than 1 kb against the National Center for Biotechnology Information (NCBI) database. In addition, a pan-genome matrix was built using Roary with default options (https://sanger-pathogens.github.io/Roary/), and gene content was visualized with Phandango (https://jameshadfield.github.io/phandango/).

### Functional annotation of genes

Because the reference strains NCTC8325 and N315 presented 54 and 25% of their coding sequences (CDSs) annotated as “hypothetical proteins,” we reannotated the genes identified in our analysis using InterProScan (www.ebi.ac.uk/interpro/) and BLAST searches against the Conserved Domain Database (www.ncbi.nlm.nih.gov/Structure/cdd/cdd.shtml) or Pfam protein families database (https://pfam.xfam.org/). In addition, we assigned clusters of orthologous groups to mutated genes using the eggnog mapper (*58*).

### Phylogenetic and population genetic analysis

To infer the isolate genealogies and branch lengths, we manually constructed core genome SNP alignments from the variants characterized and used them to build minimum evolution phylogenetic trees using the maximum composite likelihood method in MEGA (Molecular Evolutionary Genetics Analysis) (*59*). This method was used because of the short evolutionary frames and previous knowledge on the transmission chains. From the variants identified, we estimated the substitution rates as described in Eq. 1: number of mutations (*m*) divided by the genome size (*N*) times the generations (*t*/*g*). Considering a replication time of 30 min for *S. aureus*, 1 year is approximately equivalent to 18,000 generations.

$$\mu =\frac{m}{N*(\frac{t}{g})}$$ (1)

To examine within-host dynamics, we calculated the total number of SNPs per isolate, as well as fixed and variable SNPs present in each of the three isolates from individual sheep. We applied a general linear regression model on SNP counts versus number of days to estimate differences between the molecular clocks for transmissions and within single host population dynamics. Pairwise genetic distances were calculated as the number of SNPs between two isolates from the same host.

### Bacterial genome evolution simulations

We simulated the evolution of genome populations using GenomePop2 (*35*), a forward time simulation tool. A limitation of these algorithms is the exponential increase of computational intensiveness with genomic length. Thus, we only simulated 1% of the typical *S. aureus* genome, i.e., 28 kb, specifying a mutation rate of 0.0001 SNPs per genome per generation (around 3.5 × 10^−9^ mutations per base per generation) and with recombination set to 0. Assuming a generation time of 30 min and an experiment run for 400 days, we simulated 17,281 generations, with a maximum population size of 1.25 × 10^6^ individuals, which is equivalent to 5000 CFU/ml in a maximum volume of 250 ml. To examine the effect of different types of bottlenecks, we simulated a constant population size, tight bottlenecks produced by transmissions, wide bottlenecks produced by lambs feeding, and a combination of both. In addition, we simulated evolving populations under two selection models: lack of selection (all neutral mutations) and a hypothetical distribution with selection (fig. S1). This distribution was based on previous research (*36*, *60*), and because we were simulating adaptation to a new niche, most mutations were set as lethal, deleterious (gamma distribution) and neutral, and very few mutations slightly beneficial. In total, we simulated eight scenarios and ran 100 replicates for each one. We sampled 1000 genomes every 1000 generations and estimated a number of population parameters, including the genetic diversity (as mean pairwise number of SNPs between samples), fixed number of SNPs, variable number of SNPs, and frequency and variation of genotypes.

### Genetic modifications

General DNA manipulations were performed using standard procedures. Plasmid constructs used in this study were generated by cloning PCR products obtained with the oligonucleotide primers indicated in table S8. To introduce restriction sites in strains NCTC8325 and N315, we used plasmids pMAD and pBT2_bgaB and the oligonucleotides listed in table S8. pJP1501 and pJP1502 were constructed by a two-piece overlap assembly PCR. The whole fragments were subsequently cloned into the pMAD and pBT2_bgaB vectors, and the plasmids were transformed by electroporation into *S. aureus* RN4220 and transduced into NCTC8325 and N315 strains. Allelic replacements were carried out by a two-step procedure as follows: First, the plasmids were integrated into the chromosome by homologous recombination under nonpermissive conditions (44°C) and subsequent growth of the cointegrates at permissive temperature (30°C) followed by a second homologous recombination under nonpermissive conditions (44°C), resulting in their resolution. Erythromycin (for pMAD)– and chloramphenicol (for pBT2_bgaB)–sensitive white colonies, which no longer contained the pMAD or pBT2_bgaB plasmid, were tested by PCR. To differentiate between wild-type (wt) and mutant strains, we performed PCRs with oligonucleotides indicated in table S8 and digestion with restriction enzymes specific for Eco RI in NCTC8325s strain or Hind III for N315s isolates. All mutations were confirmed by DNA sequencing.

### Temporal identification of the appearance and fixation of mutations

The time of acquisition and fixation of the nonsynonymous SNP present in locus *aacA* (encoding the acetyl-CoA carboxylase α subunit) was identified by selecting 10 colonies of NCTC8325s (NCTC8325 vWbp_EcoRI) during every week after the coinoculation with NCTC8325 and NCTC8325s and performing sequencing of the PCR products obtained with primers SNP_aacA_1m to SNP_aacA_2c. To identify the time of fixation of the SNP present in 3% of the population, we extracted DNA from 400 colonies isolated from milk samples at days 14, 22, and 29 and performed PCRs to obtain an amplicon of pool DNA with primers 3SNP_hp_1m to 3SNP_hp_2c. These primers contained overhang adapter sequences that were necessary for subsequent sequencing by Illumina (MiSeq Nano 2 × 250 bp). Mapping to the reference genome permitted to identify the counts present in nucleotide 401 corresponding to SNP present in 3% of the population.

### Statistical analyses

Statistical analyses were performed with R version 3.3.2 for one-tailed Fisher’s exact test and Barnard’s test in the coinfection experiments comparing the number of sheep infected by the wt or evolved strains. For the comparison of the evolutionary dynamics of the within-host and transmitted isolates, linear models were fitted and analysis of covariance (ANCOVA) tests were performed on the mutation rates and intercepts of regression lines using type of isolates as covariate.

### Ethics statement

Experiments were conducted in accordance with paragraph 2a of article 10 of Decree 13/2007 of 26 January of the Regional Government, on the protection of animals used for experimentation and other scientific purposes in the Valencia Region (DOCV no. 5439 of 30.01.2007) establishing the functions of the Autonomous Commission for Animal Welfare in Experimental Animals. In addition, experiments were conducted in accordance with the European principles regarding the protection of animals used for experimental and other scientific purposes (Council Directive 86/609/EEC).

## Supplementary Material

http://advances.sciencemag.org/cgi/content/full/5/11/eaax0063/DC1

Download PDF

Table S1

Table S2

A multihost bacterial pathogen overcomes continuous population bottlenecks to adapt to new host species

## SUPPLEMENTARY MATERIALS

Supplementary material for this article is available at http://advances.sciencemag.org/cgi/content/full/5/11/eaax0063/DC1 Fig. S1. Distribution of selection coefficients in the computer simulations of evolving populations. Fig. S2. Growth curves in ewe milk. Table S1. Detailed transmission chains of the infections. Table S2. Information on the isolates used in this study. Table S3. Remaining mutations acquired during the infections and passages. Table S4. Coinfection experiment results. Table S5. Coinfection experiment results with isogenic strains. Table S6. SNP fixed in the population at different times. Table S7. Counts of the SNP found in 3% of the population. Table S8. Bacterial strains, plasmids, and oligonucleotides used in this study.

## Appendix

View/request a protocol for this paper from *Bio-protocol*.
