# Supplementary material for: A multihost bacterial pathogen overcomes continuous population bottlenecks to adapt to new host species
Source: Sci Adv. 2019 Nov 27;5(11):eaax0063. doi: 10.1126/sciadv.aax0063 (PMC6881152; doi:10.1126/sciadv.aax0063)
Supplement: http://advances.sciencemag.org/cgi/content/full/5/11/eaax0063/DC1 [file supp_5_11_eaax0063__index.html]

Science Advances | Science AdvancesAAASSearchScience AdvancesMenu

## Supplementary Materials

**The PDFset includes:**

- Fig. S1. Distribution of selection coefficients in the computer simulations of evolving populations.
- Fig. S2. Growth curves in ewe milk.
- Legends for tables S1 and S2.
- Table S3. Remaining mutations acquired during the infections and passages.
- Table S4. Coinfection experiment results.
- Table S5. Coinfection experiment results with isogenic strains.
- Table S6. SNP fixed in the population at different times.
- Table S7. Counts of the SNP found in 3% of the population.
- Table S8. Bacterial strains, plasmids, and oligonucleotides used in this study.

Download PDF

**Other Supplementary Material for this manuscript includes the following:**

- Table S1 (Microsoft Excel format). Detailed transmission chains of the infections.
- Table S2 (Microsoft Excel format). Information on the isolates used in this study.

**Files in this Data Supplement:**

- Adobe PDF - aax0063\_SM.pdf
