# Supplementary material for: A multihost bacterial pathogen overcomes continuous population bottlenecks to adapt to new host species
Source: Sci Adv. 2019 Nov 27;5(11):eaax0063. doi: 10.1126/sciadv.aax0063 (PMC6881152; doi:10.1126/sciadv.aax0063)
Supplement: Download PDF [file aax0063_SM.pdf]

Science Advances
AAAS

advances.sciencemag.org/cgi/content/full/5/11/eaax0063/DC1

Supplementary Materials for

A multihost bacterial pathogen overcomes continuous population bottlenecks to adapt to new host species

Rodrigo Bacigalupe, María Ángeles Tormo-Mas, José R. Penadés*, J. Ross Fitzgerald*

*Corresponding author. Email: ross.fitzgerald@ed.ac.uk (J.R.F.); joser.penades@glasgow.ac.uk (J.R.P.)

Published 27 November 2019, *Sci. Adv.* **5**, eaax0063 (2019)
DOI: 10.1126/sciadv.aax0063

The PDF file includes:

Fig. S1. Distribution of selection coefficients in the computer simulations of evolving populations.
Fig. S2. Growth curves in ewe milk.
Legends for tables S1 and S2.
Table S3. Remaining mutations acquired during the infections and passages.
Table S4. Coinfection experiment results.
Table S5. Coinfection experiment results with isogenic strains.
Table S6. SNP fixed in the population at different times.
Table S7. Counts of the SNP found in 3% of the population.
Table S8. Bacterial strains, plasmids, and oligonucleotides used in this study.

Other Supplementary Material for this manuscript includes the following:

(available at advances.sciencemag.org/cgi/content/full/5/11/eaax0063/DC1)

Table S1 (Microsoft Excel format). Detailed transmission chains of the infections.
Table S2 (Microsoft Excel format). Information on the isolates used in this study.

Supplementary Materials

Animals employed in the study.

Healthy sheep (species*: Ovis aries*; race: aragonesa; sex: female; age: 3-6 years old; weight: 50-60 kg) were obtained from commercial livestock (Fabregat y Saura, CB) in Guadalajara (Spain). Transport complied with the Council Regulation (EC) No 1/2005 on the protection of animals during transport and related operations. Upon arrival at our facilities, animals were housed in random groups and their diet consisted of alfalfa hay.

Bacterial growth

The bacterial strains used in this study are listed in the Table S8. *S. aureus* strains were cultured at 37 °C in tryptic soy broth (TSB) or agar (TSA) plates supplemented with erythromycin (10 μg/ml, Sigma-Aldrich) or chloramphenicol (20 μg/ml, Sigma-Aldrich) as needed. *Escherichia coli* was grown at 37 °C in Luria-Bertani broth (LB) supplemented with ampicillin (100 μg/ml, Sigma-Aldrich) as appropriate.

Intra-mammary infections

Prior to infection, sheep were isolated from lambs in different pens for 2-3 h. At the time of infection, the teat was sterilized with 70% ethanol. The first jets of milk were discarded before a sterile cannula (Bovivet) was used to introduce 1 ml of PBS with the required number of bacteria via the teat, before carefully removing the cannula. Holding the opening of the teat to keep it closed, it was massaged to move the inoculum further into the gland and prevent it from escaping. To prevent suckling leading to possible elimination of the infection, lambs were kept separately from their mothers until 4 h post-innoculation.

Sampling of ewe's milk.

The lambs were separated 2 to 3 h before sampling to facilitate milking, and samples were obtained in sterile tubes and plated onto blood agar plates within 2 h. In cases where the samples had a very high bacterial count, the milk was diluted to obtain isolated colonies. Milk samples were stored frozen in 15% (v/v) glycerol.

Preparation of inoculum for ewes.

In the case of the initial infection, a 1:50 dilution from an overnight culture was made in sterile TSB and incubated at 37 ºC with shaking at 120 rpm for 2 h reaching an O.D. of 0.3-0.4. 2 ml of the culture were collected and centrifuged at 3500 r.p.m. for 5 min. The cells were suspended and washed twice with PBS, re-suspended in PBS and OD600 measured. The number of bacteria present in the resuspension was estimated, considering that 1 OD600 = 4.9E8 CFU of *S. aureus*. Serial dilutions were made in PBS to obtain the desired CFU/ml (40 CFU/ml). Before infecting, 100 µl was plated onto TSA plates to ensure that the infection was carried out with the appropriate size inoculum. In the case of coinfections, the wt and passaged strains were prepared independently and mixed at the time of dilution.

For subsequent infections, milk taken from ewes was plated onto blood agar plates and 50 to 100 colonies were collected and suspended in PBS, followed by resuspension and dilution to the desired inoculum size as described previously.

Supplementary Figures

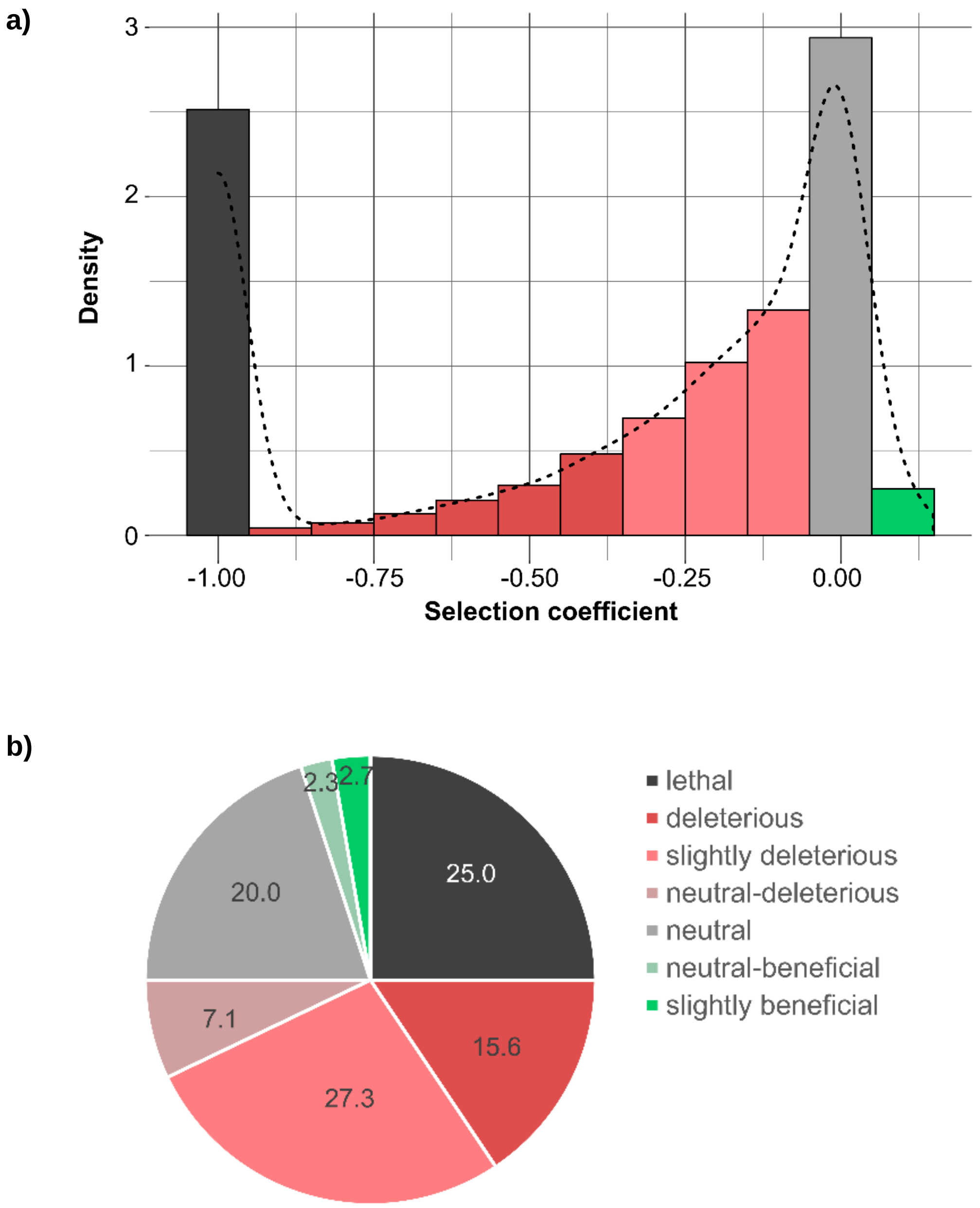

Fig. S1. Distribution of selection coefficients in the computer simulations of evolving populations. Distribution of selection coefficients, with deleterious mutations following a gamma distribution (a). Pie chart showing the proportions of different selection coefficients (b).

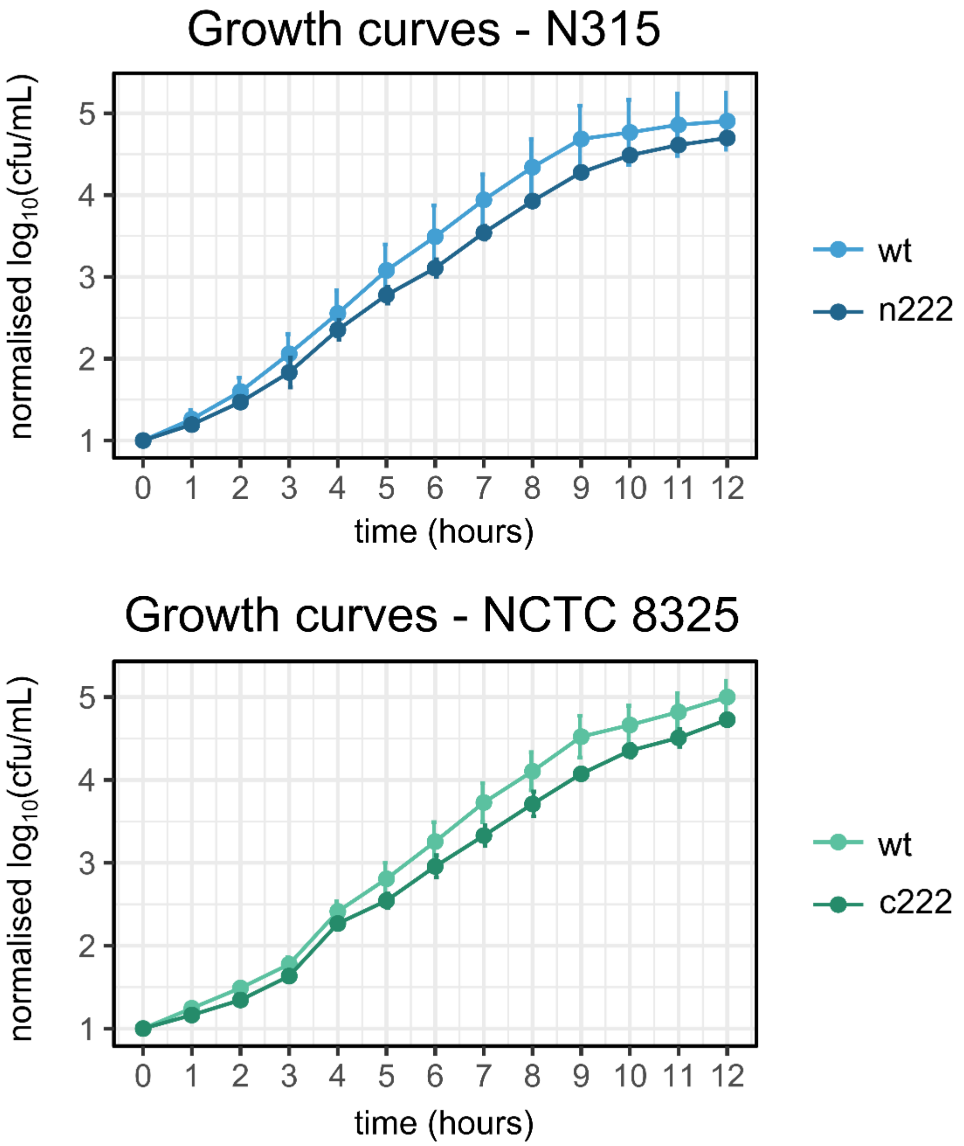

Fig. S2. Growth curves in ewe milk. a) Comparison between the wt strain N315 and its corresponding passaged strain c222. b) Comparison between the wt strain NCTC8325 and its corresponding passaged strain n222.

Table S1. Detailed transmission chains of the infections. Information on the transmission chains carried out in the experimental design, indicating the number of sheep inoculated in every passage, the number of infected sheep and the names of the corresponding sheep (in blue).

Table S2. Information on the isolates used in this study. Description of all the isolates used for the inoculations, *in vitro* experiments and those obtained from the sheep passages. Table includes information on the strain (NCTC8325 or N315), name of isolate in the laboratory, clonal sub-lineage, the animal it was recovered from, animal inoculated, number of days it had been passaged in total and number of days it was in each animal.

Table S3. Remaining mutations acquired during the infections and passages.

Strain	Mutation	bp	Type	Gene
**NCTC8325**	SNP	229054	Synonymous variant	SAOUHSC 00206
**NCTC8325**	SNP	272032	Synonymous variant	SAOUHSC 00253
**NCTC8325**	SNP	385688	Intergenic	-
**NCTC8325**	SNP	422373	Intergenic	-
**NCTC8325**	SNP	559526	Intergenic	-
**NCTC8325**	SNP	585234	Intergenic	-
**NCTC8325**	SNP	623832	Intergenic	-
**NCTC8325**	SNP	627083	Intergenic	-
**NCTC8325**	SNP	636338	Synonymous variant	SAOUHSC 00647
**NCTC8325**	SNP	799564	Synonymous variant	SAOUHSC 00817
**NCTC8325**	SNP	1081697	Synonymous variant	SAOUHSC 01128
**NCTC8325**	SNP	1286784	Intergenic	-
**NCTC8325**	SNP	1340783	Synonymous variant	SAOUHSC 01398
**NCTC8325**	SNP	1355452	Intergenic	-
**NCTC8325**	SNP	1377147	Synonymous variant	SAOUHSC 01447
**NCTC8325**	SNP	1684494	Synonymous variant	SAOUHSC 01787
**NCTC8325**	SNP	1719769	Synonymous variant	SAOUHSC 01813
**NCTC8325**	SNP	1904942	Intragenic variant	SAOUHSC R0003
**NCTC8325**	SNP	1906596	Intergenic	-
**NCTC8325**	SNP	2083424	Intergenic	-
**NCTC8325**	SNP	2093784	Intergenic	-
**NCTC8325**	SNP	2242811	Intragenic variant	SAOUHSC R0005
**NCTC8325**	SNP	2268784	Synonymous variant	SAOUHSC 02444
**NCTC8325**	SNP	2451999	Intergenic	-
**NCTC8325**	SNP	2452011	Intergenic	-
**NCTC8325**	SNP	2452012	Intergenic	-
**NCTC8325**	SNP	2711148	Synonymous variant	SAOUHSC 02947
**NCTC8325**	Del	224013	Intergenic	-
**NCTC8325**	Del	396980	Intergenic	-
**NCTC8325**	Del	1021508	Intergenic	-
**NCTC8325**	Del	1461353	Intergenic	-
**NCTC8325**	Del	1776477	Intergenic	-
**NCTC8325**	Ins	2032782	Intergenic	-
**NCTC8325**	Ins	2448064	Intergenic	-
**NCTC8325**	Del	2525646	Intergenic	-
**N315**	SNP	53449	Intergenic	-
**N315**	SNP	353405	Intergenic	-
**N315**	SNP	365241	Intergenic	-
**N315**	SNP	476211	Intergenic	-
**N315**	SNP	524171	Intergenic	-
**N315**	SNP	586838	Synonymous variant	SA RS02935
**N315**	SNP	777422	Intergenic	-
**N315**	SNP	1129690	Synonymous variant	SA RS05640
**N315**	SNP	1155166	Synonymous variant	SA RS05815
**N315**	SNP	1191837	Synonymous variant	SA RS05980
**N315**	SNP	1555299	Intergenic	-
**N315**	SNP	1882251	Intragenic variant	SA RS09295
**N315**	SNP	1987904	Synonymous variant	SA RS09960
**N315**	SNP	2386274	Synonymous variant	SA RS12190
**N315**	SNP	2408705	Synonymous variant	SA RS12305
**N315**	SNP	2625804	Intergenic	-
**N315**	Del	297980	Intergenic	-
**N315**	Del	604199	Intergenic	-
**N315**	Del	2031700	Intergenic	-
**N315**	Del	2376037	Intergenic	-

Mutations with low or modifier effects (synonymous or intergenic variants) are listed. Single-nucleotide polymorphisms (SNP), deletions (Del) and insertions (Ins).

Table S4. Coinfection experiment results.

Progenitor	Passaged	Sheep	Bacteria recovered	Progenitor	Passaged	Sheep	Bacteria recovered
NCTC8325	C421	OVC421.1	passaged	N315	N1122	N1122.1	progenitor
		OVC421.2	passaged			N1122.2	passaged
		OVC421.3	passaged			N1122.3	passaged
		OVC421.4	progenitor			N1122.4	progenitor
		OVC421.5	passaged			N1122.5	progenitor
		OVC421.6	progenitor			N1122.6	progenitor
		OVC421.7	passaged			N1122.7	passaged
		OVC421.8	progenitor			N1122.8	passaged
		OVC421.9	progenitor	N315	N222	N222.1	passaged
		OVC421.10	passaged			N222.2	passaged
		OVC421.11	passaged			N222.3	passaged
NCTC8325	C221	OVC221.1	passaged			N222.4	passaged
		OVC221.2	passaged			N222.5	progenitor
		OVC221.3	progenitor			N222.6	passaged
		OVC221.4	passaged			N222.7	passaged
		OVC221.5	passaged			N222.8	passaged
		OVC221.6	passaged			N222.9	progenitor
		OVC221.7	passaged			N222.10	passaged
		OVC221.8	passaged				
		OVC221.9	passaged				
		OVC221.10	passaged				

After co-infection with progenitor and passaged strains (NCTC8325s/C421, NCTC8325s/C221, N315s/N1122, N315s/N222), we differentiated between both strains by PCR of 10 colonies with oligonucleotides indicated in the Table S8 and digestion with *Eco*RI for NCTC8325s or *Hin*dIII for N315s. The results after 40 d post co-inoculation are indicated. Co-infection NCTC8325s/C421: 4 ewes all colonies corresponding with progenitor strain and 7 ewes with all colonies corresponding to passaged strain; co-infection NCTC8325s/C221: 1 ewe with progenitor strain and 9 with passaged strain; co-infection N315s/N1122: 4 ewes with progenitor strain and 4 with passaged strain; co-infection N315s/N222: 2 ewes with progenitor strain and 8 with passaged strain.

Table S5. Coinfection experiment results with isogenic strains.

Sheep	Day 1			Day 8			Day 14			Day 22			Day 29			Day 40		
		PCR			PCR			PCR			PCR			PCR			PCR	
		wt	eco		wt	eco		wt	eco		wt	eco		wt	eco		wt	eco
**1I**	10000	8	11	250	0	10	100	0	10	0	-	-						
**8I**	0	-	-	0	-	-	0	-	-	0	-	-						
**9I**	300	13	5	10000	0	10	10000	1	9	*	0	6	5000	0	10	10000	0	10
**14I**	10000	8	8	2000	0	10	10000	0	10	200	0	8						
**16D**	5000	12	8	9	8	0	5000	10	0	30	10	0						
**18I**	*	6	12	40	0	10	600	0	10	300	0	9						
**19I**	100	19	1	50	8	0	100	9	0	50	8	0						
**28I**	300	1	19	0	-	-	0	-	-	0	-	-						
**30I**	300	20	0	7	5	0	0	-	-	0	-	-						
**33I**	30	9	2	50	0	7	1000	0	10	*	0	8						
**2I**	30	8	5	1	0	1	0	-	-	0	-	-						
**15I**	200	0	20	*	0	9	5000	0	9	8000	0	8						
**17D**	30	14	4	20	10	0	100	10	0	0	-	-						
**20I**	0	-	-	0	-	-	0	-	-	0	-	-						
**22D**	2000	20	0	*	10	0	300	10	0	200	10	0						
**24D**	30	20	0	300	10	0	1000	9	0	3000	9	0						
**32D**	100	11	9	1000	10	0	0	-	-	x								
**38D**	300	11	9	0	-	-	0	-	-	0	-	-						
**41D**	*	8	12	50	6	3	80	9	1	500	9	1	800	10	0	10000	10	0
**21I**	0	-	-	0	-	-	0	-	-	0	-	-						

After co-infection with the wt (NCTC8325) and the isogenic (NCTC8325s; eco) strains, we differentiated them by PCR with the oligonucleotides indicated in the Table S8 and digestion with *Eco*RI. The table contains the number of CFU/ml in the milk sampled on different days as counted on blood agar plates (* indicates very high concentration of bacteria, as lawns were observed on the plates).

Table S6. SNP fixed in the population at different times.

Day post-inoculation	wt	SNP fixed
1	10	0
7	0	10
14	0	10
21	0	10
27	0	10
34	0	10

The table indicates the counts of colonies with the SNP fixed. Sequencing results of the PCR products corresponding with the locus *aac*A with primers SNP_*aac*A_1m – SNP_*aac*A_2c. We performed a PCR of 10 colonies of NTCT8325s at different time-points post-infection of sheep 9I with strains NTCT8325 and NTCT8325s. The sequencing results show that in the first day post-infection all colonies were wt but from the end of the first week post-infection, all isolates contained the SNP in locus *aac*A.

Table S7. Counts of the SNP found in 3% of the population.

Days post infection	Total Counts	A counts	% A	C counts	% C	G counts	% G	T counts	% T
15	60664	8	0%	60592	100%	3	0%	25	0%
22	50211	9	0%	49960	100%	1	0%	214	0%
29	50048	11	0%	49989	100%	1	0%	28	0%

This mutation is a missense variant in a hypothetical protein. The table indicates the percentages of nucleotides counts. Results of Miseq Illumina sequence of amplicon obtained by PCR of a DNA pool with primers 3SNP_hp_1m - 3SNP_hp_2c. DNA from 400 CFU isolated from milk samples at days 14, 22 and 29 was pooled, amplified and sequenced. The table includes total counts, counts of each nucleotide individually and percentages for each nucleotide.

Table S8. Bacterial strains, plasmids, and oligonucleotides used in this study.

Resource	Source	Identifier
**Bacterial and Virus Strains**		
Laboratory strain, restriction-defective derivate of RN450	Kreiswirth *et al.*, 1983	RN4220
Human *S. aureus* strain. HG001 (NTC8325 *rsbU* repaired)	Herbert *et al.*, 2010	NCTC8325
Human *S. aureus* strain	Kuroda *et al.*, 2001	N315
NCTC8325 with restriction site *Eco*RI in *vWbp*	This paper	JP10586
N315 with restriction site *Eco*RI in *ArlR*	This paper	JP10775
**Recombinant DNA**		
Vector for efficient allelic replacement	Arnaud *et al.*, 2004	pMAD
Vector for efficient allelic replacement	Bruckner *et al.*, 1997	pBT2
pMAD restriction_site_*Eco*RI_vWbp8325	This paper	pJP1501
pBT2_ bgaB restriction_site_*Hin*dIII_ArlRN315	This paper	pJP1502
**Oligonucleotides**		
SNPfixed_aacA: GGTAAAACCCAGTATCGATT	This paper	SNP_aacA_1m
SNPfixed_aacA: TGGAAAGACAGTAATTTGGC	This paper	SNP_aacA_1m
SNP3%_hp: TCGTCGGCAGCGTCAGATGTGT ATAAGAGACAGAATAAATCAAGTATTAACTA GTCACGGAAGGTAG	This paper	3SNP_hp_1m
SNP3%_hp: GTCTCGTGGGCTCGGAGATGTGT ATAAGAGACAGGGGTGAAATTCAATGATTGGA CAAAC	This paper	3SNP_hp_2c
pJP1501: ACGCGTCGACGGGATTATTAGCATCA ATAGG	This paper	vWbp8325-1mS
pJP1501: CAATTTCTTTTAAGAATTCCTCACTTTG TTTTTTC	This paper	vWbp8325-2c
pJP1501: GAAAAAACAAAGTGAGGAATTCTTAAA AGAAATTG	This paper	vWbp8325-3m
pJP1501: CGCGGATCCATTTGTTGCTGAGTTT GACGC	This paper	vWbp8325-4cB
vWbp_*Eco*R1: GGTTTCTGGGGAGAAGAATCC	This paper	vWbp8325-5m
vWbp_*Eco*R1: TCTAAAACAGGAATATTATTAGG	This paper	vWbp8325-6c
pJP1502: CGCGGATCC GCAATCAGAATTAAC AGAATGC	This paper	ArlRN315-1mB
pJP1502: CGATCGTATGGTTTAAGCTTGTTT CGTAAATATC	This paper	ArlRN315-2c
pJP1502: GATATTTACGAAACAAGCTTAAA CCATACGATCG	This paper	ArlRN315-3m
pJP1502: ACGCGTCGAC GAATAATTTGTAA TGGTGTTCG	This paper	ArlRN315-4cS
ArlR_*Hin*dIII: GGATATTATCGATGTCAACGG	This paper	ArlRN315-5m
ArlR_*Hin*dIII: CAAAATAACGGTGTTCATAACC	This paper	ArlRN315-6c
